# Salt-bridge modulates differential calcium-mediated ligand binding to integrin α1- and α2-I domains

**DOI:** 10.1038/s41598-018-21231-1

**Published:** 2018-02-13

**Authors:** Kyle L. Brown, Surajit Banerjee, Andrew Feigley, Hanna Abe, Timothy S. Blackwell, Ambra Pozzi, Billy G. Hudson, Roy Zent

**Affiliations:** 1 Department of Medicine, Vanderbilt University Medical Center, Nashville, TN 37232–2372 USA; 2 Center for Structural Biology, Vanderbilt University Medical Center, Nashville, TN 37232–2372 USA; 3 Center for Matrix Biology, Vanderbilt University Medical Center, Nashville, TN 37232–2372 USA; 4 Department of Chemistry and Chemical Biology, Cornell University, Ithaca, NY 14853 USA; 5 Northeastern Collaborative Access Team, Argonne National Laboratory, Lemont, IL 60439 USA; 6 Leadership Alliance, Vanderbilt University Medical Center, Nashville, TN 37232–2372 USA; 7 Aspirnaut Summer research program, Vanderbilt University Medical Center, Nashville, TN 37232–2372 USA; 8 Veterans Affairs Hospital, Nashville, TN 37232 USA; 9 Department of Biochemistry, Vanderbilt University, Nashville, TN 37232–2372 USA

## Abstract

Integrins are transmembrane cell-extracellular matrix adhesion receptors that impact many cellular functions. A subgroup of integrins contain an inserted (I) domain within the α–subunits (αI) that mediate ligand recognition where function is contingent on binding a divalent cation at the metal ion dependent adhesion site (MIDAS). Ca^2+^ is reported to promote α1I but inhibit α2I ligand binding. We co-crystallized individual I-domains with MIDAS-bound Ca^2+^ and report structures at 1.4 and 2.15 Å resolution, respectively. Both structures are in the “closed” ligand binding conformation where Ca^2+^ induces minimal global structural changes. Comparisons with Mg^2+^-bound structures reveal Mg^2+^ and Ca^2+^ bind α1I in a manner sufficient to promote ligand binding. In contrast, Ca^2+^ is displaced in the α2I domain MIDAS by 1.4 Å relative to Mg^2+^ and unable to directly coordinate all MIDAS residues. We identified an E152-R192 salt bridge hypothesized to limit the flexibility of the α2I MIDAS, thus, reducing Ca^2+^ binding. A α2I E152A construct resulted in a 10,000-fold increase in Mg^2+^ and Ca^2+^ binding affinity while increasing binding to collagen ligands 20%. These data indicate the E152-R192 salt bridge is a key distinction in the molecular mechanism of differential ion binding of these two I domains.

## Introduction

**Figure 1:**
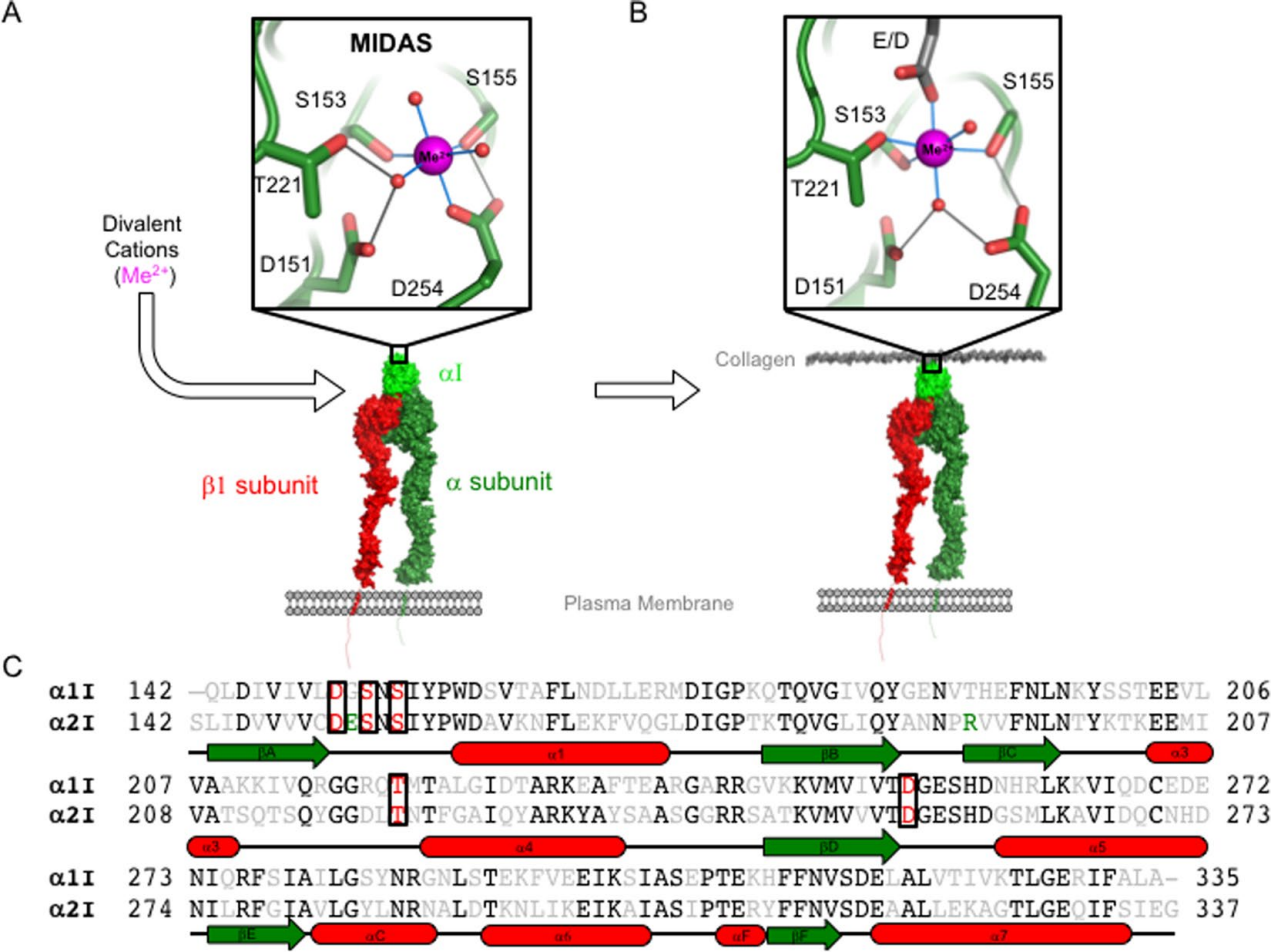
The integrin α1I and α2I MIDAS is central to ligand binding. A model of integrin subdomain architecture illustrates that the MIDAS of the integrin αI domain (αI) is central to ligand binding. Divalent metals (Me^2+^) activate the I domain by binding to the MIDAS in a “closed” conformation (**A**). These ions then form an essential force-bearing metal-protein bond with acidic residues of ligand molecules (**B**). The I domain MIDAS shifts to an “open” conformation upon interaction with ligands of the ECM, e.g. collagen, and initiates signal transduction to intercellular machinery. Primary sequence alignment of α1I and α2I illustrates secondary structural elements with an overall 52% sequence identity (**C**). MIDAS residues are highlighted in red, conserved residues are in black, and non-conserved residues are in grey. The R192-E152 salt bridge of α2I is highlighted in green.

Integrins are widely expressed cell surface receptors that couple cell- extracellular matrix (ECM) interactions with the cytoskeleton and transduce mechanochemical signals across the plasma membrane initiating a biological response. Integrins comprise different combinations of non-covalently linked α and β subunits that determine ligand specificity and function^1^. Of the 24 known mammalian integrin heterodimers, 9 contain an inserted domain within the extracellular domain of the α-subunit (αI, Fig. 1A)^2,3^. I domain-containing integrins are important therapeutic targets in inflammation, transplantation, and autoimmunity^4–6^ and are highly conserved, originating in early chordates^7^. Four I domain-containing integrins (α1β1, α2β1, α10β1, and α11β1) function as collagen receptors on numerous cell types. The remaining five, integrins αDβ2, αLβ2, αMβ2, αXβ2, αEβ7, are exclusive to leukocytes.

Integrin function is dependent on the interplay between divalent cation cofactors, particularly Mg^2+^ and Ca^2+^^8^. For example, an essential feature of the integrin activation mechanism is a force-bearing metal bond linking integrin α-subunit I domains (αI) with physiological ligands (Fig. 1B)^9^. The bond consists of a glutamate or aspartate in the ligand and a metal ion bound by a metal ion dependent adhesion site (MIDAS) within the I domain. The MIDAS contains conserved residues located in three loops on one surface of the I domain. Non-conserved residues surrounding the MIDAS contribute to ligand specificity (Fig. 1C)^10–21^. Metal binding to the integrin β-subunit is required for allosteric signal transduction where the Mg^2**+**^-binding “I-like” domain MIDAS is flanked on either side by Ca^2+^-binding adjacent to MIDAS (ADMIDAS) and synergistic metal-binding sites (SYMBS)^22–26^. The combination of metal binding motifs with their discrete metal preferences across integrin subunits set up a complex interplay necessary for integrin function^27,28^. Reductionist approaches have revealed contrasting roles for Mg^2+^ and Ca^2+^ in integrin α1-I domain (α1I) and integrin α2-I domain (α2I) ligand binding. Specifically, Mg^2+^ facilitates binding to both α1I and α2I. In contrast, while Ca^2+^ promotes α1I ligand binding, it inhibits α2I binding^29^, suggesting these ions differentially regulate the interaction of integrins with their cognate ligands.

Integrin I domains fully preserve the ligand-binding functionality of full-length integrins^10,29–33^. This makes them simple and convenient models for analysis of integrin-ligand binding mechanisms and deciphering metal-dependent properties without the complications introduced by interdependencies of multiple metal-binding sites present in full-length or ectodomain integrin constructs. I domains contain approximately 200 amino acids that assume a Rossmann fold where a central β-sheet is surrounded by amphipathic α-helices^2^. I domains are reported to exist in either an open, closed, or intermediate conformation where ligand affinity is greatest in the open conformation^2,3,21,34–39^. Mutagenesis studies have demonstrated the impact of I domain modifications on overall integrin activation, ligand binding, and subsequent function^40–43^. This study focuses on the highly homologous collagen-binding α1I and α2I (Fig. 1C). The seminal crystal structure of the α2I in complex with a GFOGER triple-helical mimetic peptide^44^ and a NMR-refined model of α1I in complex with a GLOGEN triple-helical mimetic peptide^21^ have provided significant insight into collagen ligand recognition and suggest a common mode of activation. Yet, α1I and α2I have a differential response to select metal cofactors whose mechanism of action remains unknown.

Despite the contrasting role of Ca^2+^ in mediating I domain function, to date, the molecular mechanism by which Ca^2+^ selectively activates α1I and deactivates α2I is unknown. We hypothesize that differential Ca^2+^-MIDAS association facilitates α1I activation, but prohibits α2I activation, thus affecting subsequent ligand binding. In the present study, X-ray crystallography in combination with molecular modeling, genetic sequence analysis, and isothermal calorimetry were used to delineate the molecular mechanisms that modulate Ca^2+^-dependent α1I and α2I ligand binding. I domains were co-crystallized with Ca^2+^ to gain an atomic level comparison of Ca^2+^-MIDAS interactions. Cross comparisons with previously determined Mg^2+^-bound I domain structures reveal that α1I binds both Mg^2+^ and Ca^2+^ in a manner sufficient to promote ligand binding. In contrast, Ca^2+^ is displaced in the α2I MIDAS relative to Mg^2+^ and is unable to fully coordinate MIDAS residues. Crystallographic B-factors suggest reduced MIDAS loop flexibility prohibit α2I from fully accommodating Ca^2+^. We determined that disruption of a non-conserved E152-R192 salt bridge within α2I increased Ca^2+^ binding, indicating that it functioned, in part, to constrict α2I MIDAS flexibility. These findings identify a novel mechanism of differential I domain activation whereby I domain-mediated integrin α1β1 and α2β1 signaling is potentially regulated.

## Results

### Structure of the I domains

**Figure 2:**
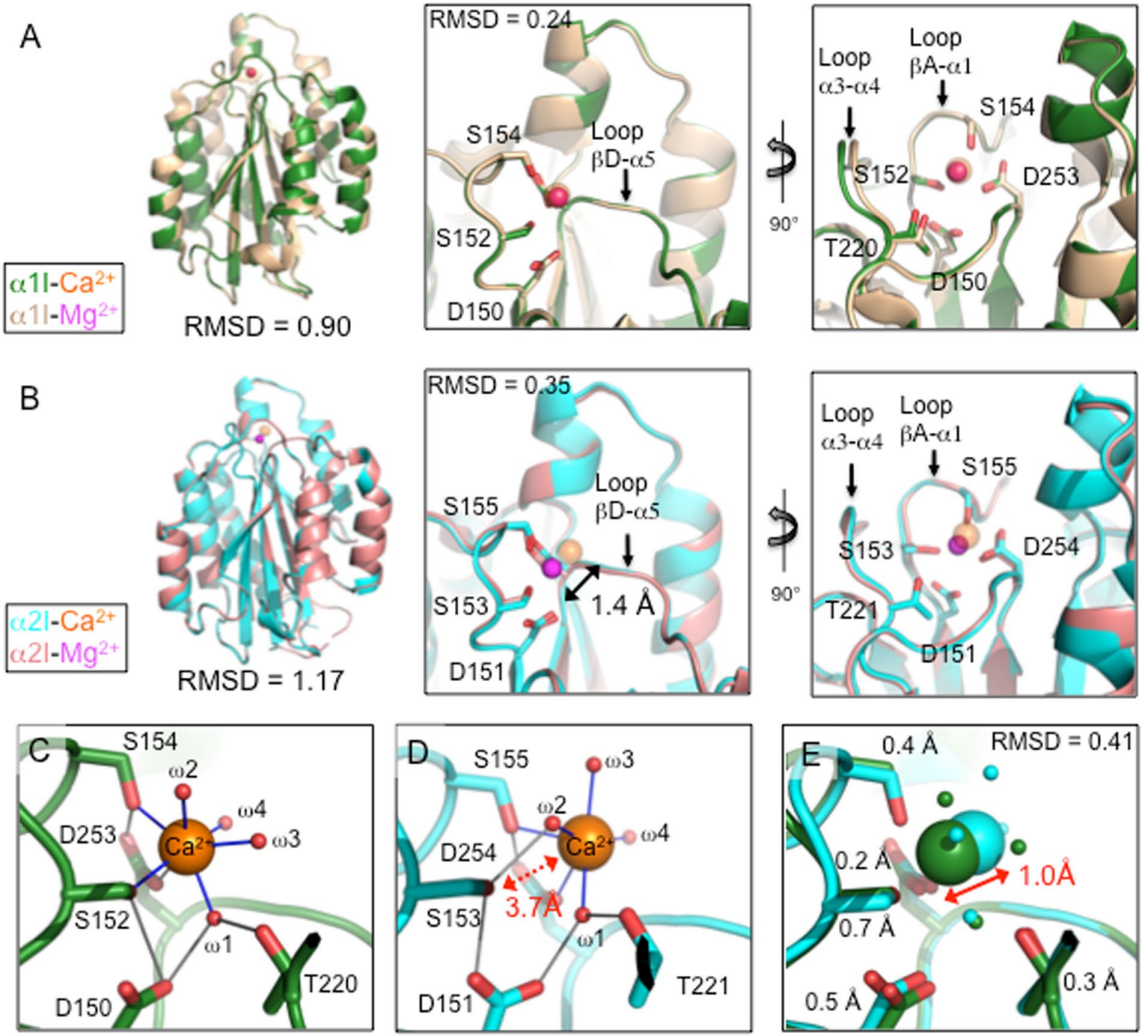
Ca^2+^ vs. Mg^2+^-bound I domain structural comparison reveal Ca^2+^ displacement in the α2I. Tertiary structural comparison of the Mg^2+^-bound α1I (1QCY, light brown) and the Ca^2+^-bound α1I (5HGJ, dark green) produced a heavy atom RMSD of 0.90 Å. Locations of the MIDAS-bound Ca^2+^ and Mg^2+^ were superimposable. Ca^2+^ ions are depicted as orange spheres while Mg^2+^ ions are depicted as magenta spheres (magenta appear red when overlaid with orange Ca^2+^). Comparison of α1I Mg^2+^-bound MIDAS residues and Ca^2+^-bound MIDAS residues produced a heavy atom RMSD of 0.24 Å (**A**). Structural comparison of the Mg^2+^-bound α2I (1AOX, pink) and the Ca^2+^-bound α2I (5HJ2, cyan) produced a heavy atom RMSD of 1.17 Å. Comparison of α2I Mg^2+^-bound MIDAS residues and Ca^2+^-bound MIDAS residues produced a heavy atom RMSD of 0.35 Å. The Ca^2+^ ion was displaced an average of 1.4 Å out of the MIDAS pocket relative to the Mg^2+^ ion (**B**). The α1I Ca^2+^ ion coordinated residues S154, S152, D253 (monodentate) and four water molecules (blue lines). Coordination with residues D150 and T220A is water-mediated (ω1) (**C**). The α2I Ca^2+^ ion coordinated residues S155, D254 (monodentate) and four water molecules (blue lines). Coordination with residues S153, D151 and T221 are water-mediated (ω1 & ω2). An average distance of 3.7 Å prohibits direct S153 coordination with the Ca^2+^ ion (**D**). Structural comparison of the Ca^2+^-bound α1I and α2I MIDAS residues yield a heavy atom RMSD of 0.41 Å. Comparative residue displacements were measured from Ser OG, Thr OG1, Asp CG atoms: D150/151, 0.5 Å; S152/153, 0.7 Å; S154/155, 0.4 Å; T220/221, 0.3 Å; and D253/254, 0.2 Å (**E**). Chain A from α1I and α2I structures was used to generate figures. (See also Figs S1, S2, S3, S4, Table S1).

The α1I and α2I structures were determined at 1.4 and 2.15 Å resolution, respectively. Data processing and refinement statistics are listed in Table 1. The structures of both α1I and α2I, as expected, assume a Rossmann fold where a central hydrophobic β-sheet is surrounded by seven amphipathic α-helices (Fig. 2). Both α1I and α2I are in the “closed” ligand binding conformation. Asymmetric unit (AU) intermolecular contacts with Ca^2+^, MIDAS loops, α7 helix, or other known conformational triggers were not observed for either α1I or α2I. The α1I asymmetric unit comprises two molecules whose structures were analyzed for discrepancies relevant to Ca^2+^ binding. The RMSD between the A and B α1I molecules is 0.13 Å (Table S1). Based on 2mFo-DFC and omit map densities, water molecule positions were analogous in both α1I chains (Fig. S1). The α2I asymmetric unit comprises six molecules A through F. RMSD values range between 0.18–0.38 Å. RMSD values for α2I MIDAS residues were between 0.10–0.24 Å (Table S1). Structural measurements for α2I are reported as averages between molecules A, B, C, & D. Chains E and F were omitted from the α2I average because local MIDAS structures were deviant from canonical conformations. Specifically, chain F was omitted because Ca^2+^ and water molecules were not located in anomalous or omit maps, even in lower sigma values (Fig. S2). Chain E was omitted because Ca^2+^ was not fully hydrated resulting in coordination with S153 in lieu of water-mediated coordination with D152 and T220. Detailed measurement of individual α1I and α2I AU molecule MIDAS distances and ion coordination properties are detailed in Fig. S3. A structural alignment of Ca^2+^-bound I domains with Mg^2+^-bound I domains (α1I, 1QCY; α2I, 1AOX)^45,46^ indicated minor structural variations. Ca^2+^ vs. Mg^2+^-bound α1I have an RMSD of 0.90 Å while the α2I exhibit an RMSD of 1.17 Å (Fig. 2). Minor structural discrepancies exist between the N- and C-termini, however, these differences were not considered significant to Ca^2+^ binding.

**Table 1:** Crystallographic data processing and refinement statistics.

	α**1I**	α**2I**
**Data collection**		
PDB id	5HGJ	5HJ2
Wavelength (Å)	0.9791	0.9791
Detector	Pilatus-6M	Pilatus-6M
Resolution range^#^ (Å)	150–1.4 (1.45–1.4)	150–2.15 (2.23–2.15)
Space group	P2_1_	P4_3_2_1_2
Cell Parameters (Å,°)	37.37, 95.95, 53.08, 90.0, 104.0, 90.0	144.07, 144.07, 130.00, 90.0, 90.0, 90.0
Total reflections	243413	593410
Unique reflections	67092	73620
Multiplicity*	3.6 (2.3)	8.0 (5.0)
Completeness* (%)	94.6 (90.0)	99.3 (93.7)
Mean* I/σ(I)	14.7 (1.9)	11.6 (2.0)
Wilson B-factor	17.4	24.9
R_merge_*	9.7 (59.8)	17.9 (69.2)
**Refinement**		
R_work_/R_free_ (%)	17.7/20.6	23.3/27.3
Number of molecules per AU	2	6
Number of atoms:		
(all/protein/ions/water)	3555/3084/3/462	9496/9094/43/359
Protein residues	388	1177
r.m.s. bonds/angles(Å/o)	0.006/1.003	0.009/1.112
Ramachandran favored/outliers (%)	98.0/0.0	98.0/0.3
B-factor (all/protein/ions/solvent)	19.6/18.2/15.3/28.9	34.3/34.3/47.9/31.6

^#^Highest resolution shell in parentheses.

^*^Data in parentheses are calculated for the highest-resolution shell.

### Identity of crystallographic metal cofactors

I domains were co-crystalized with Ca^2+^ salts; however, their incorporation into the structures was not assured. Therefore, crystallographic ions found at the MIDAS where identified by two methods. First, anomalous difference maps were calculated using data collected above the absorption edge of Ca^2+^. Strong anomalous densities at the MIDAS are coincident with a Ca^2+^ ion in both α1I and α2I structures (Fig. S4A,B). Second, energy-dispersive X-ray spectroscopy (EDS) analysis was used to unequivocally identify the metals found in α1I and α2I crystals (Fig. S4C,D). Fluorescent emission peaks at 2.61 keV and 2.82 keV are consistent with the Kα1 and Kβ1 of chloride, while emission peaks at 3.70 keV and 4.01 keV are consistent with the Kα1 and Kβ1 of calcium, respectively. Collectively these data indicate Ca^2+^ ions are present in α1I and α2I crystals and located at the MIDAS.

### Structure of the MIDAS

Integrin I domains have been the subject of numerous structural studies that provide a basis to compare Ca^2+^-bound structures^2,9,21,39,40,42,45,47–50^. Specifically, it is known the metal ion in the MIDAS is coordinated by side chains from three loops on the upper surface of the I domains. Loop βA-α1 contains the conserved DxSxS sequence (residues 150–154, α1I; 151–155, α2I) (Fig. 2). Loop βD-α5 contains D253 in α1I and the analogous D254 in α2I. Loop α3-α4 contains T220 in α1I and the analogous T221 in α2I that participates in a water-mediated interaction to the MIDAS metal in the absence of ligand.

In the current study, we observed the positions of the Ca^2+^, the DxSxS MIDAS motif, and the C-terminal α7-helix indicate that both I domains are in the closed, low affinity ligand binding conformation analogous to those of other isolated I domain structures^2,9,21,39,40,42,45,47–50^. More specifically, the α1I domain Ca^2+^ coordinates D253, S152 and S154 (Fig. 2A). D150 and T220 interactions with Ca^2+^ are water-mediated (ω1, Fig. 2C). In total, the Ca^2+^ is heptacoordinated with three residues and four water molecules. The metal-I domain side chain bond distances are approximately 2.4 Å. The position of the Ca^2+^ ion is superimposable when compared to Mg^2+^ (1QCY). Exclusive alignment of Ca^2+^ vs. Mg^2+^-bound α1I MIDAS residues produced a RMSD of 0.24 Å. The main chain position of loops βA-α1 and βD-α5 was superimposable. In contrast, loop α3-α4 was displaced away from the MIDAS.

In comparison, α2I revealed Ca^2+^ coordinates D254 and S155, but not S153 directly. Ca^2+^ coordination with D151, T221, and S153 (Figs 2D, S3) are water-mediated. An average of two residues and four water molecules coordinated the Ca^2+^ ion within the AU. In comparison with the Mg^2+^ structure the ions exhibit distinct differences. A 1.4 Å shift of Ca^2+^ from the Mg^2+^ position produces an average distance of 3.7 Å from S153, a distance insufficient to facilitate direct hydrogen bonding. Residue S153 was displaced an average of 0.7 Å away from the metal while the hydroxyl of S155 was positioned 0.4 Å further into the MIDAS pocket sterically obstructing Ca^2+^ access (Fig. 2E). The main chain position of loops βA-α1, βD-α5, and α3-α4 was analogous to the Mg^2+^-bound structure. Alignment of Ca^2+^-bound Chain A vs. Mg^2+^-bound α2I MIDAS residues produced a RMSD of 0.35 Å. Alignment of Ca^2+^-bound α1I and α2I MIDAS residues produced a RMSD of ~0.4 Å. Comparison of Ca^2+^ positions indicate the α2I ion is displaced ~1.0 Å further into solvent than the α1I Ca^2+^. Electrostatic surface potentials of α1I and α2I show a large electronegative patch dominates the MIDAS face. Significant differences in electrostatic topology were not apparent suggesting differential Ca^2+^ binding is not a product of divergent surface charge distribution (Fig. S5).

### Mechanism of differential Ca^2+^ binding

**Figure 3:**
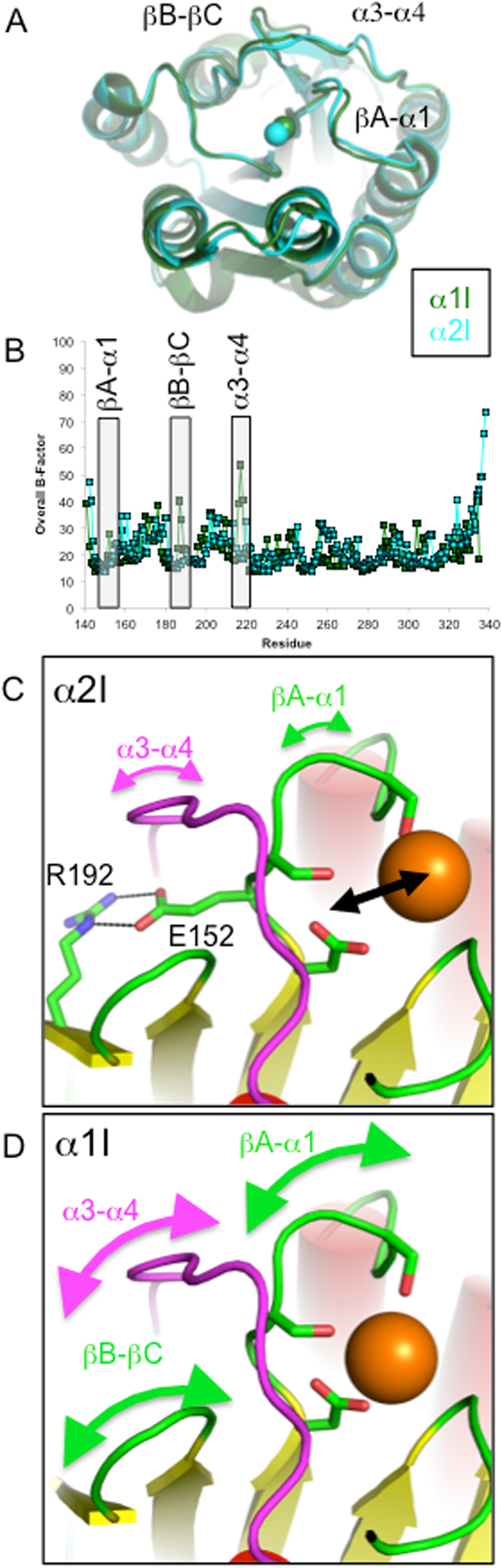
I domain loop regions adjacent to the MIDAS are more flexible in α1I than α2I. Top view of the MIDAS face of the Ca^2+^-bound α1I (dark green) and the α2I (cyan) indicates expansion of α1I MIDAS loops allow for better accommodation of larger Ca^2+^ ions (RMSD 1.21 Å) (**A**). Relative differences in residue-averaged B-factors suggest distinct regions of thermal motion in α1I; loops α3-α4, βA-α1, and βB-βC (boxed) (**B**). Molecular modeling suggests the R192-E152 salt bridge in α2I functionally limits MIDAS residue flexibility. (**C**) This salt bridge is not conserved in the α1I structure. (**D**) (See also Fig. S5).

The α1I and α2I structures were analyzed for properties that could cause differential Ca^2+^ MIDAS binding. The largest structural distinctions between α1I and α2I occur at loops αC-α6, βA-α1, βB-βC, and α3-α4 (Fig. 3A). Significantly, loops α3-α4, βA-α1, and βB-βC comprises MIDAS residues or residues that maintain contact with MIDAS residues. Temperature factor (B-factor) analysis was used to investigate the potential of contrasting loop movement (Fig. 3B). B-factors are flexibility reporters measuring the degree of isotropic smearing of electron density, reflecting differences in structural dynamics^51^. Average B-factors had significant contributions from both main chain and side chain B-factors (Fig. S5). Overall, both I domains exhibit a fairly rigid structure with the largest degrees of motion limited to loop regions. Comparison of Ca^2+^-bound structures indicates the α3-α4 and βB-βC loops, and to a lesser extent the βA-α1 loop, forms a discrete pocket of motion in the α1I structure whereas the α2-I domain MIDAS face dynamics are comparatively more rigid. This observation predicts distinct patterns of thermal motion between I domains that would produce variant ΔS and ΔH terms upon Ca^2+^ binding.

**Figure 4:**
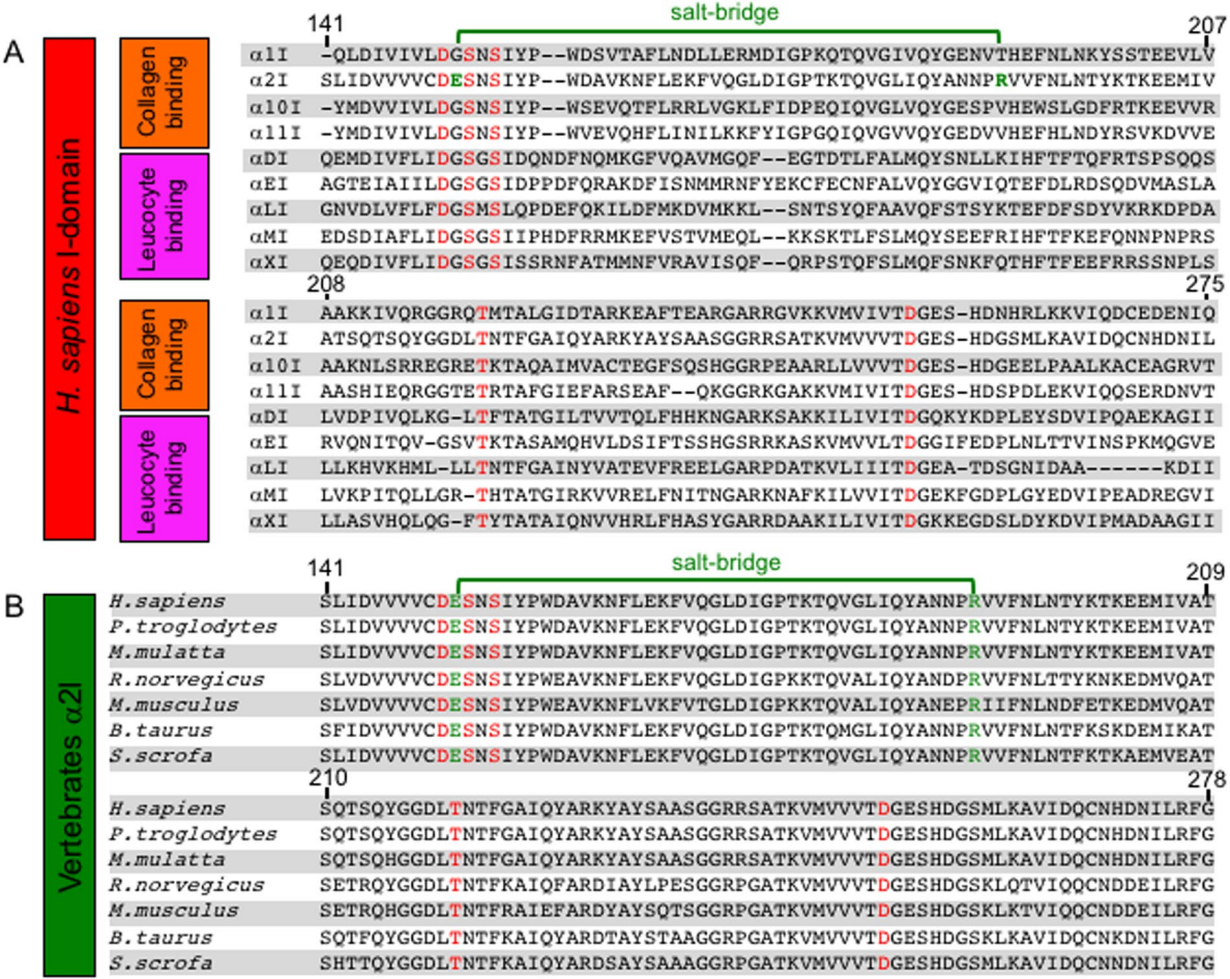
The E152-R192 salt bridge is unique within integrin α2. Multiple sequence alignment of *H. sapiens* integrin I domain sequences illustrate that MIDAS residues (red) are conserved across I domain containing integrins. (**A**) In contrast, the E152-R-192 salt bridge is unique to the α2I (green). Multiple sequence alignment of I domain primary sequences of higher vertebrates indicates both MIDAS residues (red) and E152-R192 salt bridge residues (green) are conserved across species.

To further investigate the origin of varied MIDAS loop motion in I domain structures, molecular modeling and primary sequence analysis was used to identify non-conserved residues that likely contribute to differential Ca^2+^ binding. We observed unique intra-chain polar contacts unique to each I domain. However, the α2I R192-E152 salt bridge was the most likely candidate to affect MIDAS flexibility (Figs 1C, 3C). The E152 is located between MIDAS contact residues D151 and S153 effectively tethering these residues to β-sheet C of the Rossmann fold core. Thus, the R192-E152 salt bridge could function as a brace decreasing motion in residues of the βB-βC and βA-α1 loops of α2I restricting access to the MIDAS by comparatively large Ca^2+^ ions. It is plausible that steric or electrostatic interactions in turn limit α3-α4 loop motion. Primary sequence analysis of integrin I domains reveal that while MIDAS residues are conserved across both collagen- and leucocyte-binding I domains, the R192-E152 salt bridge is unique to α2I (Fig. 4A). Further analysis indicates the R192-E152 salt bridge is conserved in α2I of higher vertebrates (Fig. 4B). The absence of this salt bridge in α1I (Fig. 3D) may explain the increase in localized thermal motion observed in crystal structures. To test this hypothesis, we generated an α2I E152A mutant for binding analyses.

### Metal binding thermodynamics

**Figure 5:**
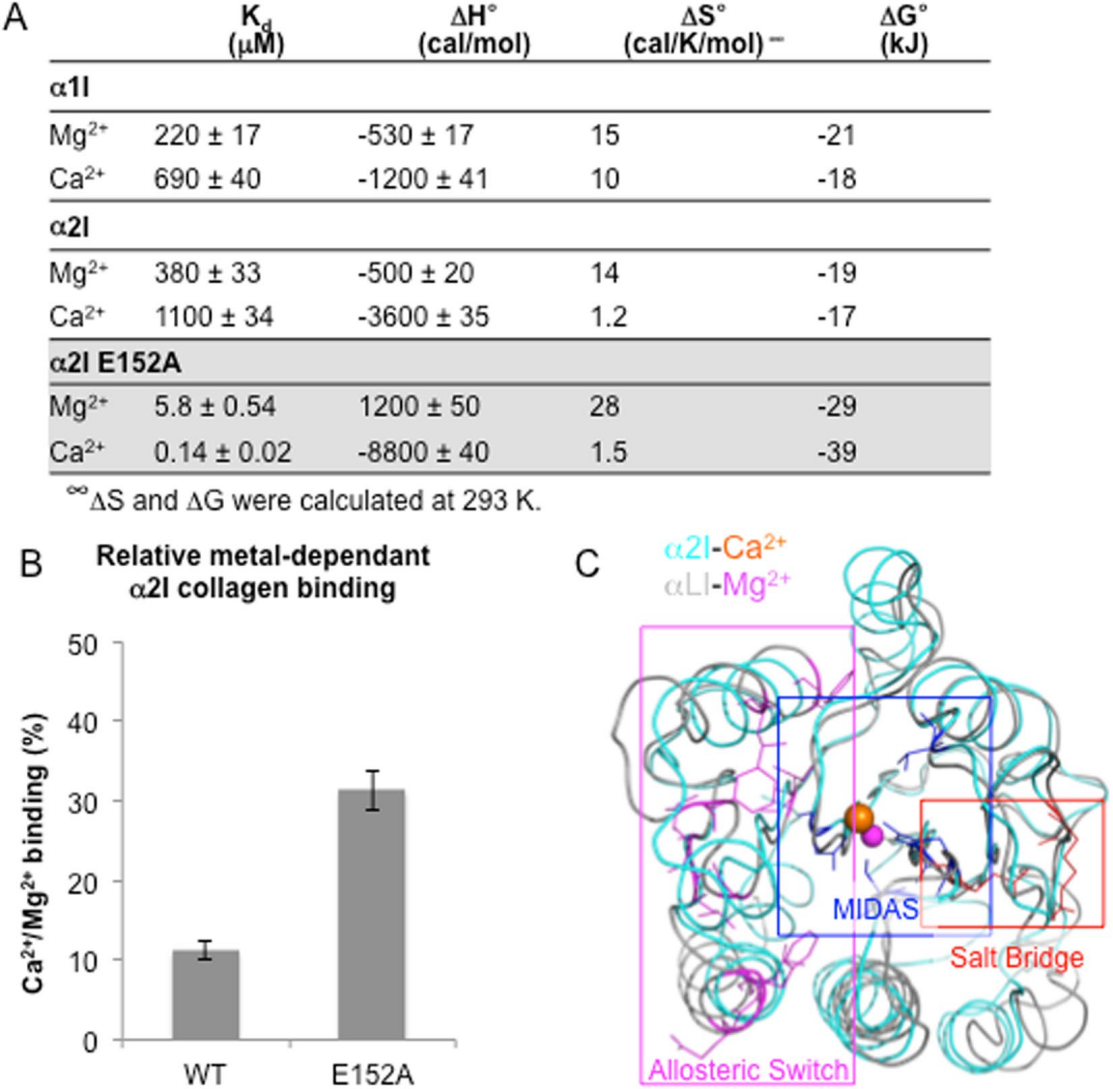
Disruption of α2I E152-R192 salt bridge alters metal binding thermodynamics and subsequent ligand binding affinity. The thermodynamics of Ca^2+^ and Mg^2+^ binding to recombinant WT I domains and the α2I E152A mutant were measured by ITC. (**A**) The impact of the α2I E152A mutation on metal-mediated binding to collagen ligands was measured by solid-phase binding assays. (**B**) Data are reported as percent increase in Ca^2+^-mediated binding relative to Mg^2+^ (Data are mean ± SE; n = 8) (See also Fig. S6) Structural alignment of αLI and α2I (RMSD 1.5 Å) indicate the α2I E152-R192 salt bridge is not directly coupled to residues involved in the allosteric mechanism of αLI activation (**C**).

Isothermal titration calorimetry (ITC) was used to quantify the heat flow associated with Ca^2+^ and Mg^2+^ binding to α1I, α2I, and the α2I E152A mutant. Figure 5A summarizes the average best-fit K_ITC_ and ΔH_ITC_ parameters from reproducible data sets. Both Ca^2+^ and Mg^2+^ titrations to either wild type I domain were exothermic. Positive ΔS° and negative ΔH° results indicate I domain metal binding is both enthalpically and entropically favored. The dissociation constants (K_d_) of Ca^2+^ and Mg^2+^ to wild type α1I and α2I are in the micromolar (μM) range at 20 °C. Comparatively lower K_d_ and ΔG° values are indicative of higher affinity of Ca^2+^ to WT α1I domain than α2I. In contrast, Mg^2+^ bound to both WT I domains with higher affinity than Ca^2+^. Ca^2+^ binding to both I domains is more enthalpically favorable than Mg^2+^ binding. While Ca^2+^ binding to WT α2I was 3-fold more enthalpically favorable than binding to α1I, Ca^2+^ binding to α1I was 8-fold more entropically favorable.

To test the hypothesis that the α2I R192-E152 salt bridge is responsible for differential metal binding properties of α1I and α2I, the binding thermodynamics of a α2I E152A mutant were compared with those of wild type. Mg^2+^ and Ca^2+^ bound α2I E152A with approximately 50-fold and 6000-fold greater affinity than WT I domains, respectively. While Ca^2+^ binding was both enthalpically and entropically favored, Mg^2+^ binding was enthalpically disfavored. Ca^2+^ binding to α2I E152A was more entropically favorable than to either WT I domain. The divergent ΔH and ΔS terms are indicative of unique static features as well as varied degrees of conformational freedom of each I domain that facilitate differential Ca^2+^ binding. In summary, binding thermodynamics indicated structural dynamics contribute to selective Ca^2+^ binding while B-factors suggest MIDAS loops are the origin.

#### Ca^2+^-mediated collagen ligand binding

Solid-phase collagen binding experiments were used to determine the effect of Ca^2+^ and the E152A mutation on α2I-collagen binding. It was confirmed that Ca^2+^ facilitated α1I binding to collagen IV tantamount to Mg^2+^ (Fig. S6). Yet, Ca^2+^ did not promote α2I or α2I E152A binding to collagen I at levels comparable to Mg^2+^. When normalized to Mg^2+^ binding, Ca^2+^ increased α2I E152A collagen binding approximately 20% (Fig. 5B). Select mutations within the αLI are known to increase ligand binding as much as 10,000-fold by altering the allosteric coupling of the α7 helix with MIDAS residues, which improves ligand access to the ID binding surface^52^. Molecular modeling reveals that residues of the αLI allosteric switch are distal to the R192-E152 salt bridge (Fig. 5C) suggesting the E152A mutation did not decouple the MIDAS from the analogous allosteric switch in α2I.

## Discussion

### Ca^2+^ binds α1I and α2I distinctly

With the notable exceptions of integrins α1β1 and αMβ2, Ca^2+^ generally inhibits I domain ligand binding^53^. Logically, Mg^2+^ and Ca^2+^ must affect integrins via distinct chemical properties. For example, the ionic radius of Ca^2+^ is larger than Mg^2+^ by 0.3 Å (Mg^2+^, 0.65 Å; Ca^2+^, 0.99 Å^54^). The surface areas of ionic spheres at these distances are 53 Å^2^ and 72 Å^2^, respectively making the Ca^2+^ ionic sphere 26% larger than Mg^2+^. In addition Mg^2+^ prefers the octahedral coordination geometry (coordination number 6), whereas Ca^2+^ can adopt octahedral, pentagonal bipyramidal, and cubic coordination geometries (coordination numbers 6 to 8)^55^. The mechanism behind the differential Ca^2+^ binding to I domains is unknown. We analyzed the α1I, where Ca^2+^ activates ligand binding, and α2I, where it suppresses ligand binding, to explain the differential effects. We determined X-ray crystal structures of both I domains with MIDAS-bound Ca^2+^ and compared them with previously determined Mg^2+^-bound I domains. We found that Ca^2+^ functionally substitutes for Mg^2+^ in the α1I structure. Specifically, Ca^2+^ is properly positioned in α1I to facilitate binding by coordination with an acidic ligand side chain, initiating the allosteric mechanism that leads to signal transduction (Fig. 2). In contrast, Ca^2+^ coordination to the MIDAS residues in α2I is diminished, reducing the force-bearing potential of the metallic bond, resulting in a less stable platform to tether ligands. Further, variant loop dynamics on the MIDAS face likely modulate differential Ca^2+^ binding by providing flexibility to the α1I MIDAS pocket for expansion to accommodate the comparatively larger Ca^2+^ ion. The α2I R192-E152 salt bridge functions to limit MIDAS expansion and subsequent Ca^2+^ binding, thus inhibiting ligand binding (Fig. 3).

Previous studies indicate that accommodation of the larger Ca^2+^ in the MIDAS octahedral environment is thermodynamically unfavorable^56^. Molecular simulations predict that structural rearrangement of the surrounding residues in the αL (LFA-1) I domain (αLI) are necessary to accommodate the larger Ca^2+^ ion resulting in a decrease in the affinity for the natural ligand, ICAM-1. Our structural data indicates that inclusion of Ca^2+^ in α1I MIDAS does not produce large structural perturbations when compared to a Mg^2+^-bound structure (Fig. 2, RMSD 0.24 Å). Although partial binding of Ca^2+^ in the α2I MIDAS produces relatively minor structural alterations when compared to Mg^2+^-bound structure (RMSD 0.35 Å), conformational rearrangement would be required to fully accommodate Ca^2+^ in agreement with αLI calculations. Collectively these observations indicate that metal interactions reveal distinct structural differences in the MIDAS region of integrin α1I and α2I.

In a survey of Ca^2+^-binding proteins, no correlation was found between Ca^2+^ affinity and many properties of the Ca^2+^ coordination sphere, e.g. net ligand charge, number of water molecules, number of protein ligands, or number of backbone protein ligands^57^. Rather, it was concluded that more subtle forces determine protein-Ca^2+^ affinity, including polypeptide strain, binding site electrostatics, and the degree of Ca^2+^-induced conformational change. Of likely significance, comparison between crystal forms of αMI from integrin αMβ2 led to the proposal that affinity regulation occurred in part via structural distinctions in MIDAS ion coordination^34^. Considering that α1I and α2I MIDAS residues are invariant, electrostatic surface potentials are comparable, and conformational change in Ca^2+^-bound structures relative to Mg^2+^ is minimal, we conclude that the differential Ca^2+^ effect is based on the intrinsic ability of each I domain to address structural strain resulting from Ca^2+^ binding. The α2I structure supports this conclusion, i.e. the R192-E152 salt bridge limits Ca^2+^ access to the MIDAS. In contrast, the α1I MIDAS is less limited by structural strain and more easily accommodates Ca^2+^.

### Thermodynamics of metal binding

To date, determined X-ray structures indicate that metals bind closed α1I and α2I conformations, which our data supports^45,46^; ligand binding is necessary to transition to the open binding conformation. Yet, solution studies indicate metal binding events can alter I domain dynamics prior to ligand binding^58^. The thermodynamics of a binding event report on the types and numbers of interactions involved in the process. When ΔH is negative, binding is enthalpically favored. Favorable enthalpy requires correct placement of hydrogen bond acceptor and donor groups at the binding interface. ΔH reflects the strength of the metal-I domain interaction relative to those with solvent, primarily due to H-bond formation and van der Waals interactions. Our data indicates that Ca^2+^ binding to α1I, α2I, and α2I E152A is more enthalpically favorable compared to Mg^2+^ binding, likely a result of its coordination flexibility (Fig. 5). Further, Ca^2+^ binding to α2I and α2I E152A is primarily enthalpically driven in comparison to the entropic term (ΔS). Positive ΔS results indicate entropically favorable metal binding. Favorable entropy changes can be due to hydrophobic interactions, specifically an increase in solvent entropy from burial of hydrophobic groups and release of water from the MIDAS upon metal binding or an increase in conformational degrees of freedom. Our data indicates that Ca^2+^ binding is comparable to Mg^2+^ binding in terms of ΔS within α1I. In contrast ΔS is approximately 8-fold less favorable with α2I, possibly a result of less efficient expulsion of water from the MIDAS or more likely a loss in conformational freedom upon Ca^2+^ binding, supporting the idea that structural dynamics are a contributing factor to selective metal binding.

Flexibility is a natural mechanism for proteins to alleviate structural strain. B-factor analysis reveals differences in loop dynamics between α1I and α2I. Specifically, increased flexibility in α1I domain loops βB-βC and α3-α4 would allow for the expansion of the MIDAS pocket necessary to bind the larger Ca^2+^ ion. This suggests differential Ca^2+^ binding of α1I and α2I and subsequent functional activation of only α1I is based on the contrasting flexibility of loops βB-βC and α3-α4 of each structure. In sum, favorable ΔS and ΔH values indicate α1I is more effective at presenting a better Ca^2+^-binding conformation than α2I while B-factors point to loops βB-βC and α3-α4 as the source of contrasting structural dynamics. The disruption of the α2I R192-E152 salt bridge was expected to increase conformational freedom as observed in the ΔS terms relative to WT α2I. However, the magnitude of divergence from WT thermodynamics was unexpected. There are multiple intra molecular non-conserved polar contacts within the α1I and α2I structures. We speculate that breaking the R192-E152 salt bridge with the α2I E152A mutant produced a domino effect, disrupting multiple non-MIDAS interactions as well.

### Impact on ligand binding

Directed evolution experiments mapped the allosteric pathway of the αLI where activating mutants increased binding affinity to physiological ligands 10,000-fold^52^. The allosteric mechanism of αLI was demonstrated to couple the MIDAS residues to the α7 helix, which controls accessing to the full I domain binding surface as well as allosteric transmission of binding signals through the integrin. The α2I E152A mutant increased Ca^2+^ binding 10,000-fold fold, yet Ca^2+^-mediated binding to collagen ligand increased approximately 20%. The apparent discrepancy in the magnitude of direct Ca^2+^ binding to α2I E152A yet relatively modest increase in Ca^2+^-mediated ligand binding suggests that the E152A mutation does not decouple access to the I domain binding surface via the analogous allosteric mechanism detailed in the αLI (Fig. 5C). Taken together with structural findings indicate that Ca^2+^ is insufficient to shift the I domain conformational equilibrium from a closed to open, high affinity binding state. Consistent with the fact that the α2I R192-E152 salt bridge does not impact ligand binding as it is intact in the open, collagen bound form of α2I^44^.

### Biological Implications

In this paper, we detail how Ca^2+^ functionally substitutes for Mg^2+^ in α1I but not in α2I where it induces a less stable ligand-binding platform. We suggest that two biological implications can be derived from these findings. First, cells invest significant energy to effect changes in Ca^2+^ concentration resulting in a gradient between their intracellular (~100 nM free) and extracellular (mM) concentrations. In contrast, the concentration of Mg^2+^ (mM) differs little across the plasma membrane^59^. Our data suggests that integrin α2β1 would be sensitive to flux in extracellular Ca^2+^ concentrations, effectively regulating ligand binding efficiency and subsequent integrin signaling. Although Mg^2+^ is not in flux, others have demonstrated that Ca^2+^ displaces I domain-bound Mg^2+^ at equimolar concentrations^58^. This data predicts that integrin α1β1 ligand binding would be comparatively less affected by Ca^2+^ concentration flux than integrin α2β1 ligand binding.

**Figure 6:**
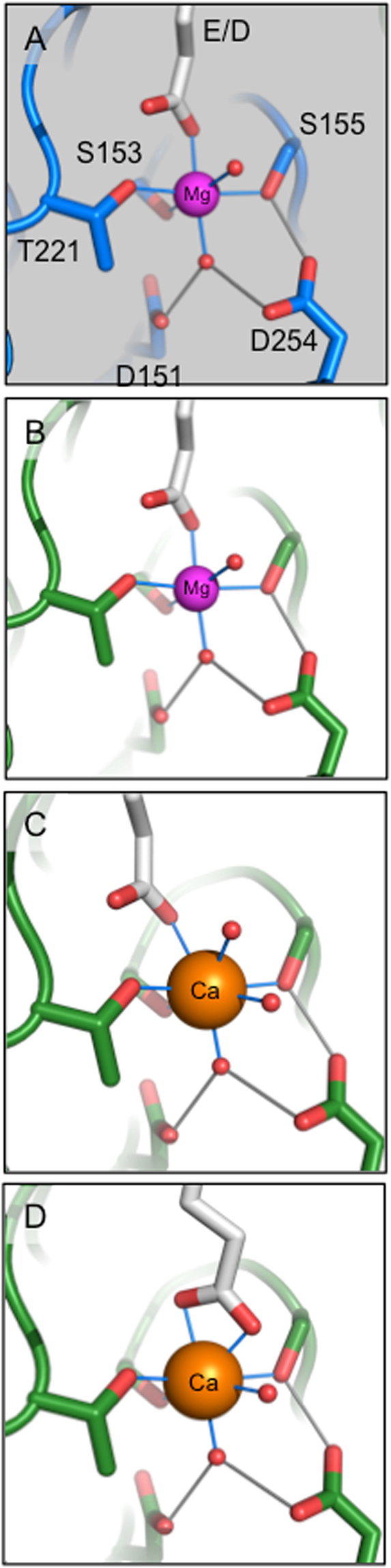
Ca^2+^ provides α1I ligand binding modes not available to α2I. Ligand binding models are based on the structure of α2I in complex with a collagen mimetic (1DZI). Mg^2+^ (magenta) coordinates MIDAS residues with an octahedral geometry. A ligand donated acidic residue (Glu or Asp) completes the coordination shell in α2I (**A**) and α1I (**B**). Ca^2+^ (orange) has flexible coordination chemistry, but prefers pentagonal bipyramidal geometry in proteins. Assuming pentagonal bipyramidal geometry, α1I can bind its ligand with either a monodentate (**C**) or bidentate (**D**) coordination.

Second, Ca^2+^ offers α1I ligand binding flexibility. Arnout *et al*. determined the crystal structures of the Fab fragment of mAb 107 complexed to the low- and high-affinity states of αMI^50^. MIDAS Ca^2+^ binding was facilitated by symmetric bidentate ligation of a Fab-derived Asp to a pentagonal bipyramidal coordinated ion. Fab fragment binding did not trigger activating tertiary changes in the αMI or in the full-length integrin. The authors determined that the denticity of the ligand Asp/Glu modified the divalent cation selection by the MIDAS and subsequent integrin function. In contrast, our data reveals the accommodation of Ca^2+^ in the α1I MIDAS is determined by intrinsic structural properties in the absence of a ligand. Significantly, the smaller Mg^2+^ ion favors monodentate binding to residues with a formal charge in an octahedral geometry, whereas the larger Ca^2+^ ion may coordinate with multiple acidic ligands often with at least one bidentate side chain. Models derived from the collagen-bound α2I (1DZI) illustrate this point (Fig. 6). Mg^2+^ can function to facilitate ligand binding in either α2I or α1I MIDAS (Fig. 6A,B). In contrast, α1I could form a monodentate bond with a ligand, assuming the preferred Ca^2+^ pentagonal bipyramidal geometry in biomolecules (Fig. 6C). In addition, Ca^2+^ can facilitate bidentate ligand binding, likely requiring some degree of conformational alteration in the ligand or I domain (Fig. 6D). Therefore, we suggest that conformational changes following Ca^2+^-mediated ligand binding may function to alter ligand selectivity. In sum, a MIDAS-bound Ca^2+^ can allow α1I to offer ligand-binding modes that Mg^2+^ cannot.

### Conclusions

These structural studies identify a novel mechanism whereby I domain-mediated integrin α1β1 and α2β1 ligand binding is differentially regulated. Specifically, the R192-E152 salt bridge prevents the α2I from using Ca^2+^ as a cofactor in ligand binding. Our findings indicate that Ca^2+^ may participate in physiological signal transduction to selectively activate integrin α1β1 over integrin α2β1 thus suggesting a potential new role for Ca^2+^ in integrin-mediated cell homeostasis.

## Materials and Methods

### Purification of I domains

Human α1I and α2I (residues 140–336)^20^ were subcloned into a pBG101 (Vanderbilt Center for Structural Biology) expression vector to produce N-terminal His6-GST fusion proteins. The fusion proteins were overexpressed in *E.coli* BL-21(DE3) cells (Novagen) in LB medium supplemented with 30 μg/ml kanamycin and 0.5 mM isopropyl-1-thio-β-D-galactopyranoside for 16–24 h at 16 °C. GST-fusion proteins were affinity purified from cell lysates with GST-sepharose beads (Thermo Scientific) and cleaved as described previously^60^. The proteins were further purified over a Superdex 200 10/300 GL size-exclusion column (GE Healthcare) equilibrated in 50 mM Tris-HCl (pH 8.0), 300 mM NaCl, 10% (v/v) glycerol, and 1 mM β-mercaptoethanol (BME). Fractions were pooled and dialyzed 1 to 1000 in 25 mM ACES or TRIS (depending on ITC or crystallography application, respectively), 100 mM NaCl, 1 mM BME, 0.01% sodium azide, and 50 mM EDTA overnight. I domains were further dialyzed 1–1000 3 times in 25 mM ACES or TRIS, 100 mM NaCl, 1 mM BME, 0.01% sodium azide and ~2 g Chelex resin (Sigma). I domain samples were analyzed for purity on 15% SDS–PAGE. Protein concentration was determined by absorbance spectroscopy at 280 nm (Nanodrop spectrophotometer 2000c) using the extinction coefficient 11460 M^−1^cm^−1^ (ProtParam^61^).

### Crystallization of I domains

Thoroughly dialyzed I domain samples were concentrated to 20–25 mg/ml. Crystals were grown by hanging drop vapor diffusion by mixing 1 μl of protein and 1–2 μl reservoir solution at 5 °C. Growth conditions for α1I were 18–26% polyethylene glycol 8 K, 100 mM Tris-HCl pH 8.0 @ RT, 20 mM CaCl_2_, 200 mM NaCl_2_, 15% glycerol. The α2I crystals grew in 16–20% polyethylene glycol 8 K, 50 mM HEPES pH 7.5, 10 mM CaCl_2_, 20% glycerol. Crystal growth was complete in 4–7 days. The approximate dimensions of α1I crystals were 200 × 50 × 50 micron while α2I crystals were 50 × 30 × 30 micron.

### X-ray Data Collection, Structure Determination, and Refinement

X-ray diffraction data were collected at beamlines 24-ID-C of the Advance Photon Source at the Argonne National Laboratory. Data were processed with HKL 2000^62^ or XDS^63^. The space groups were determined as P2_1_ and P4_3_2_1_2 for the α1I and α2I, respectively. Initial phases were determined by molecular replacement using a search model PDB id: 1QC5 for the α1I and PDB id: 1A0X, for the α2I, respectively, with Phaser^64^ in the resolution ranges of 50–1.7 Å for α1I, and 150–2.5 Å for α2I (Table 1). For α1I, two monomers were located unambiguously and refined to a final R values of R_work_ and R_free_ of 16.9 and 20.1%, respectively. For α2I, the calculated Matthew’s coefficient suggested the number of molecules in the asymmetric unit (AU) should be 6 with V_m_ = 2.5 Å^3^/Da with a solvent content of 50.7%. However, molecular replacement placed 5 molecules in the AU with partially positive densities remaining in the packing. After three cycles of rigid body refinement, the sixth molecule (Chain F) was built. The result being chains A-E are ordered and one chain (Chain F) is highly disordered. In the lattice packing the Chain F is not fully occupied in all the unit cells and the average contribution from it has poor occupancies, which precluded the placement of MIDAS Ca^2+^ and most water molecules. The phases were improved with several rounds of model building against working data sets in COOT^65^ and Phenix^66^, while 3.7% reflections (R free set) were set aside for quality control. Water molecules were adding during the last cycles of refinement. The values of the Ramachandran plot for the final refinement of the structure were obtained by the Phenix suite. The model has 1.7% outliers, mainly contributed by the Chain F. No Ca^2+^ restraints were used at any stage of refinement.

Illustrations were prepared using the coordinates of Chain A from either structure with PYMOL^67^. Electrostatic surfaces were generated with the APBS algorithm^68^. The input partial atomic charge and radius parameters were generated with the PDB2PQR^69^. Averaged B-factors were calculated with the BAVERAGE algorithm of CCP4^70^. RMSD values were calculated with PYMOL^67^.

### Accession numbers

Coordinates and structure factors have been deposited in the Protein Data Bank (www.rcsb.org) with accession number 5HGJ, α1I; 5HJ2, α2I.

### Energy-dispersive X-ray spectroscopy analysis for metal ion identity

Energy-dispersive X-ray spectroscopy (EDS) was carried out using a silicon drift detector (model X-123SDD, Amptek Inc, USA) at NE-CAT 24ID-C beam line. The built-in multi-channel analyzer of X-123SDD was calibrated with known fluorescent emission lines of multiple metals. The gain of the detector was set to 75%, corresponding to an energy range of 0–16.7 keV. EDS experiments were carried out with incident X-ray energy of 12.66 keV, just above the K absorption edge of Selenium. EDS spectrum was recorded for 60 seconds with X-rays incident on the crystal in the cryo-loop (Figs S4C and S4D).

### Isothermal calorimetry

ITC experiments were performed at 20 ± 0.2 °C on a MicroCal™ VP-ITC isothermal titration calorimeter (GE Healthcare). Solutions were degassed for 10 min prior to the experiments. Given the propensity of buffers to form metal complexes, ACES buffer (pK_a_ = 6.83, 0.1 M, 25 °C) was selected to facilitate direct comparison of I domain-metal binding results (Ca^2+^, log K = 3.38; Mg^2+^, log K = 3.55)^71^. Divalent ion sources (chloride salts of magnesium and calcium) were dissolved in buffer (25 mM ACES, pH 7.0, 100 mM NaCl, 1 mM BME, 0.01% sodium azide) and loaded into the syringe. Titrations were carried out with I domain concentrations of 100 micromolar, stirring at 307 rpm with a filter time constant of 2 s. The metal ion titrant (20 millimolar) was added in 2 microliter injections every 150 s. Blank injections of metal titrant into buffer were subtracted from individual experiments to correct for the heat of mixing and dilution. Raw data were integrated and fit with a one-site binding model to determine the best-fit values for the experimental stability constant (K_ITC_) and the change in enthalpy associated with metal binding (ΔH°) with the Microcal Origin software (GE Healthcare). Given the fitting of fewer parameters is always helpful in reducing the uncertainty^72^, the metal-I domain stoichiometry (n) was fixed at 1 based on known binding stoichiometry from structural data. Quantitative results are the average of the best-fit parameter from two or more consistent data sets. The change in free energy of each ITC titration, ΔG°, and K_d_ were determined from the equilibrium constant obtained from the best fit of experimental data, K_ITC._ Representative thermograms are included in supplemental information (Fig. S8).

### Solid-phase Collagen Binding Assays

The wells of a 96-well microtiter plate (Immulon 2, Dynatech Laboratories, Inc.) were coated overnight at 4 °C with 0.1 ml of 1 mg/ml rat tail collagen I (Corning) or mouse collagen IV (Corning) in 0.09% acetic acid. The wells were washed twice with 0.15 ml TBS and then blocked for 1 h at room temperature with 0.15 ml of 100 mg/ml bovine serum albumin (Sigma) in TBS. Recombinant GST-tagged I domains were serial diluted from 1 mM in various wash buffers (TBS containing 0.05% Tween-20, 10 mg/ml BSA, and either 5 mM EDTA, 1 mM CaCl_2_, or 1 mM MgCl_2_). The wells were washed once with 0.15 ml of the appropriate wash buffer, and then 0.1 ml of each I domain dilution was added and allowed to interact for 1.5 h at RT. Wells were washed three times with 0.15 ml of the appropriate wash buffer. Then 0.1 ml of a 1:1000 dilution of anti-GST HRP conjugate (GE Healthcare) in the appropriate wash buffer was added for 1 h at RT. Following incubation, the wells were washed three times, and then 0.06 ml of 3, 3′,5,5′-tetramethylbenzidine substrate (Sigma) was added for 1 h at RT. Reactions were stopped with 0.015 ml of 4 N H_2_SO_4_, and the plates were read at 450 nm. Representative binding plots are included in supplemental information (Fig. S8).

### Multiple Sequence Alignment

Sequences were obtained from Genbank and alignments were generated with GENEIOUS v.6.1.8 using the “blosum62” algorithm. Alignment sequences are as follows: *Homo sapiens*, ITGA1 NP_852478.1, ITGA2 NP_002194.2, ITGA10 NP_003628.2, ITGA11 NP_001004439.1, ITGAD NP_001305114.1, ITGAE NP_002199.3, ITGAL NP_002200.2, ITGAM NP_001139280.1, ITGAX NP_001273304.1; *Pan troglodytes*, ITGA2 JAA37898.1; *Macaca mulatta*, ITGA2 NP_001247751.1; *Rattus norvegicus*, ITGA2 EDM10390.1; *Mus musculus*, ITGA2 NP_032422.2; *Bos taurus*, ITGA2 NP_001159971.1; *Sus scrofa*, ITGA2 NP_001231201.1.

### Data availability

The datasets generated during and/or analyzed during the current study are available in the Protein Data Bank repository (www.rcsb.org) under accession numbers 5HGJ (α1I) and 5HJ2 (α2I).

## Electronic supplementary material

Supplementary Information
