# Supplementary material for: Salt-bridge modulates differential calcium-mediated ligand binding to integrin α1- and α2-I domains
Source: Sci Rep. 2018 Feb 13;8:2916. doi: 10.1038/s41598-018-21231-1 (PMC5811549; doi:10.1038/s41598-018-21231-1)
Supplement: Supplementary file 1 — Supplementary Information [file 41598_2018_21231_MOESM1_ESM.pdf]

Salt-bridge modulates differential calcium-mediated ligand binding to integrin α1- and α2-I domains

Kyle L. Brown[#¢∞€¥*], Surajit Banerjee[$], Andrew Feigley[¥], Hanna Abe[€], Timothy S. Blackwell[#a], Ambra Pozzi[#a∞], Billy G. Hudson[#¶♮£∞€], and Roy Zent[#a∞]

From the Departments of [#]Medicine, [¶]Biochemistry, [♮]Cell and Developmental Biology, [£]Pathology, Microbiology and Immunology, [¢]Center for Structural Biology, and [∞]Center for Matrix Biology, Vanderbilt University Medical Center, Nashville, TN 37232

[$] Department of Chemistry and Chemical Biology, Cornell University, Ithaca, NY 14853; Northeastern Collaborative Access Team, Argonne National Laboratory, Lemont, IL 60439

[a] Veterans Affairs Hospital, Nashville, TN, 37232.

[¥] Leadership Alliance research program

[€] Aspirnaut Summer research program

Supplemental Information:

A

α1I RMSD

	A	B
**A**	0.00	0.13
**B**	0.13	0.00

B

α1I MIDAS Residue RMSD

	A	B
**A**	0.00	0.00
**B**	0.00	0.00

C

Select α1I distances

	Ca vs. Mg	Ca-S152
**A**	0.1	2.6
**B**	0.1	2.6

D

α2I RMSD

	A	B	C	D	E	F
**A**	0.00	0.34	0.29	0.18	0.22	0.28
**B**	0.34	0.00	0.26	0.33	0.31	0.38
**C**	0.29	0.26	0.00	0.27	0.25	0.32
**D**	0.18	0.33	0.27	0.00	0.24	0.24
**E**	0.22	0.31	0.25	0.24	0.00	0.34
**F**	0.28	0.38	0.32	0.24	0.34	0.00

E

α2I MIDAS Residue RMSD

	A	B	C	D	E	F
**A**	0.00	0.15	0.17	0.14	0.10	0.15
**B**	0.15	0.00	0.11	0.19	0.16	0.24
**C**	0.17	0.11	0.00	0.21	0.13	0.24
**D**	0.14	0.19	0.21	0.00	0.17	0.24
**E**	0.10	0.16	0.13	0.17	0.00	0.14
**F**	0.15	0.24	0.24	0.24	0.14	0.00

F

Select α2I distances

	Ca vs. Mg	Ca-S153
**A**	1.8	4.0
**B**	1.6	3.9
**C**	1.2	3.2
**D**	1.2	3.5
**E**	0.9	2.7
**F**	NA	NA

Table S1: Asymmetric unit molecule-specific residue comparison. The α1I asymmetric unit molecules A and B were compared with respect to all atom RMSD (A) and MIDAS residues (B): L_{149}, D_{150}, G_{151}, S_{152}, N_{153}, S_{154}, I_{155} (βA-α1 loop), Q_{218}, T_{219}, M_{220} (α3-α4 loop), T_{250}, D_{251}, G_{252} (βD-α5). Ca^{2+} displacement relative to Mg^{2+} (1QCY) and the Ca^{2+} distance to S_{152}-OG were measured (C). The α2I asymmetric unit molecules A, B, C, D, E, and F were compared with respect to all atom RMSD (D) and MIDAS residues: D_{151}, E_{152}, S_{153}, N_{154}, S_{155}, I_{156} (βA-α1 loop), L_{219}, T_{220}, N_{221} (α3-α4 loop), T_{251}, D_{252}, G_{253} (βD-α5) of unit cell chains A, B, C, D, E, and F were compared with respect to all atom RMSD (including waters, E). Ca^{2+} displacement relative to Mg^{2+} (1AOX) and the Ca^{2+} distance to S_{153}-OG were measured (F).

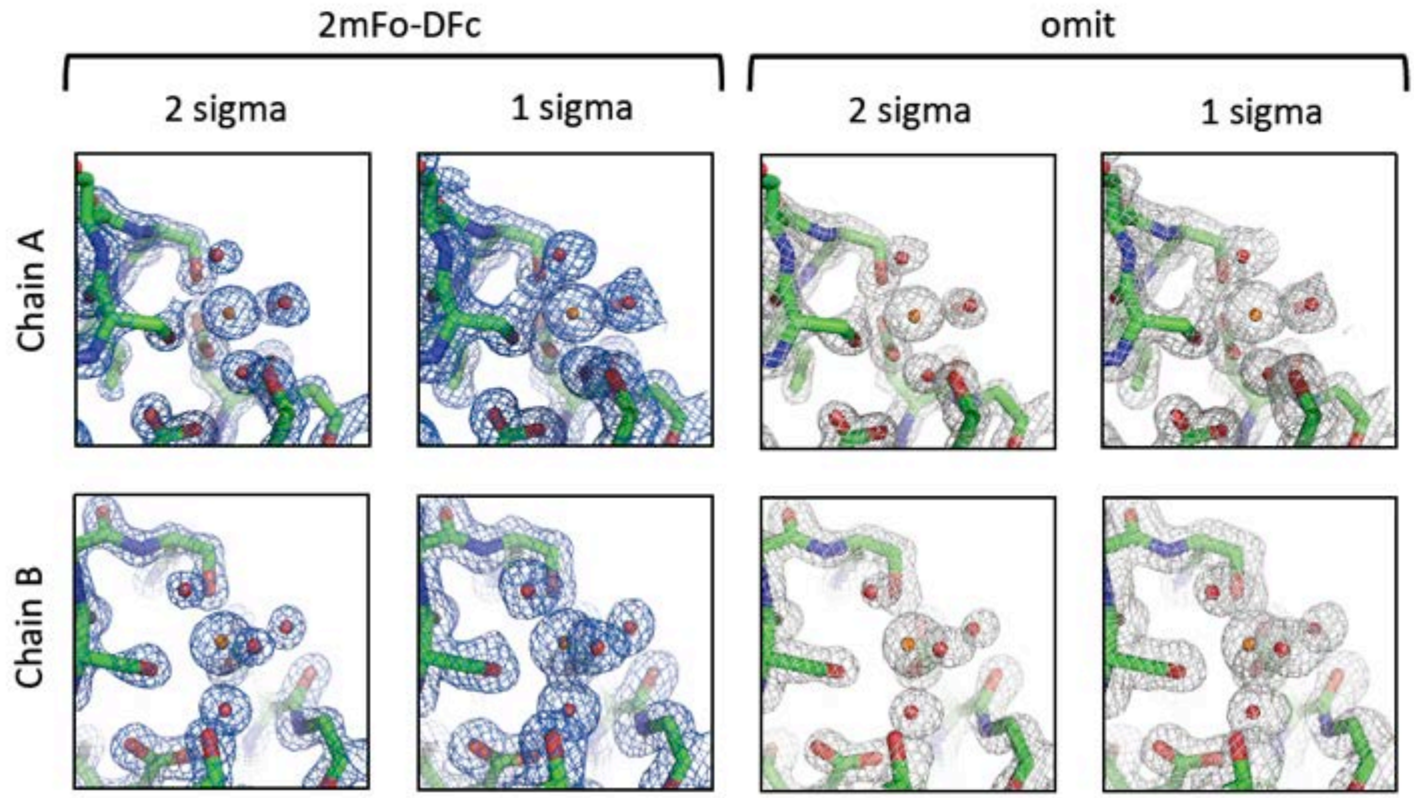

Fig. S1: Individual unit cell chain MIDAS α1I electron density maps. The 2mFo-DFC maps of α1I chains A and B are displayed in blue and the omit maps are displayed in grey at both 1 and 2 sigma. Ca^{2+} ions are depicted as orange spheres and water molecules are displayed as red spheres.

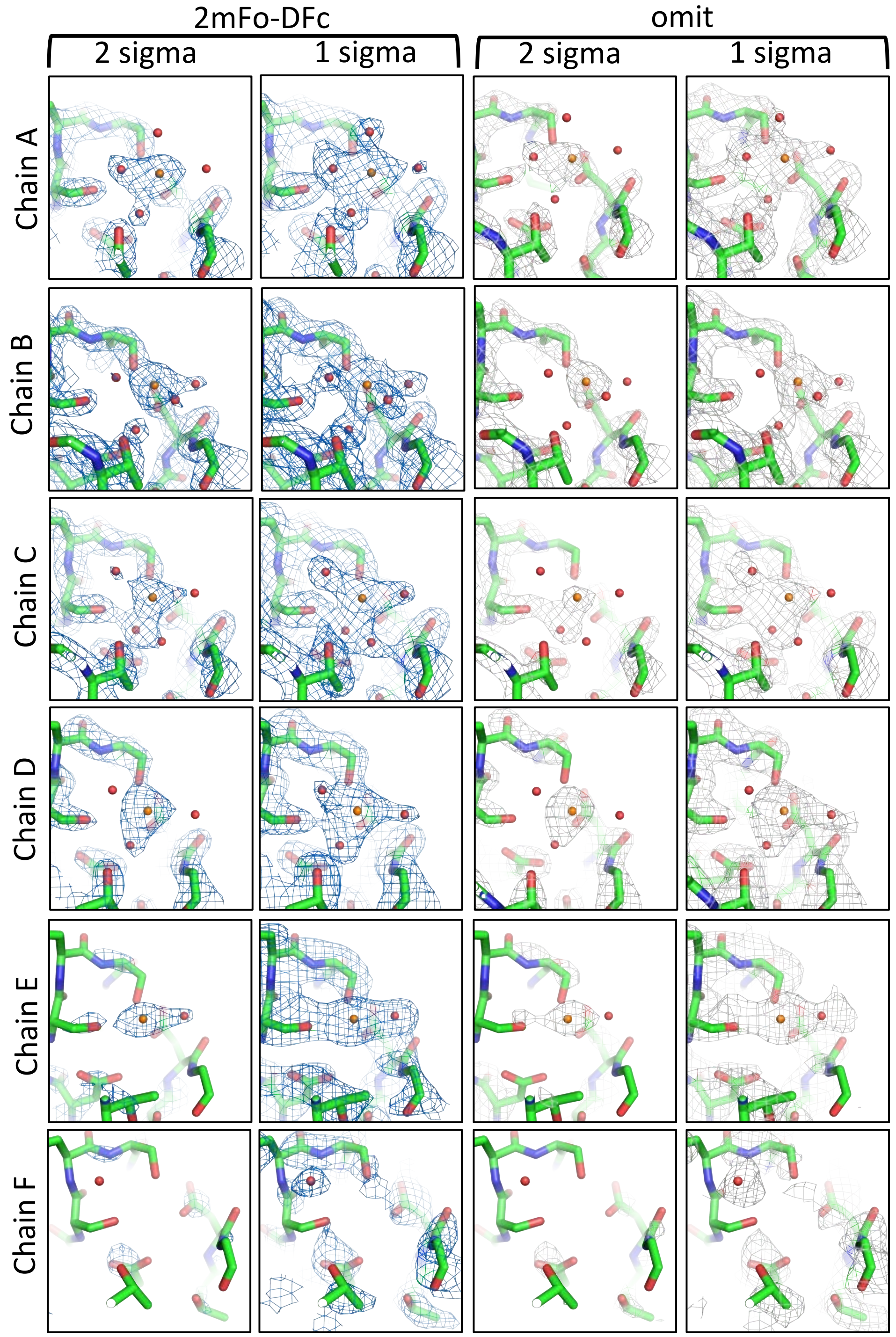

Fig. S2: Individual unit cell chain MIDAS α2I electron density maps. The 2mFo-DFC maps of α2I chains A through F are displayed in blue and the omit maps are displayed in grey. Ca^{2+} ions are depicted as orange spheres and water molecules are displayed as red spheres. Due to Chain F disorder Ca^{2+} and water molecules were not located in anomalous or omit maps, even in lower sigma values.

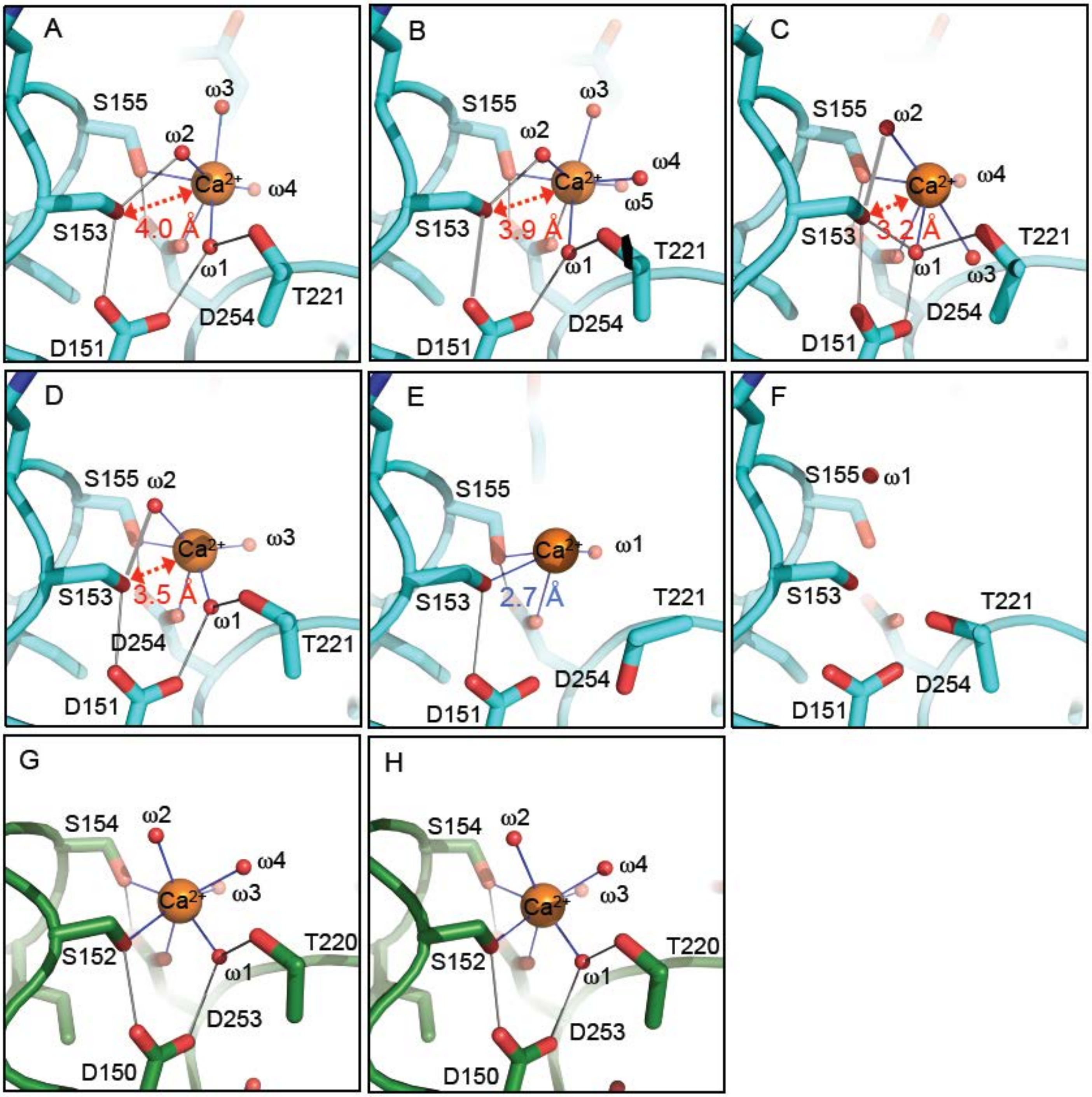

Fig. S3: Ca^{2+} coordination of individual unit cell chains. α2I chains A, B, C, D, E, F are depicted in panels A-F, respectively. α1I chains A and B are depicted in panels G and H, respectively.

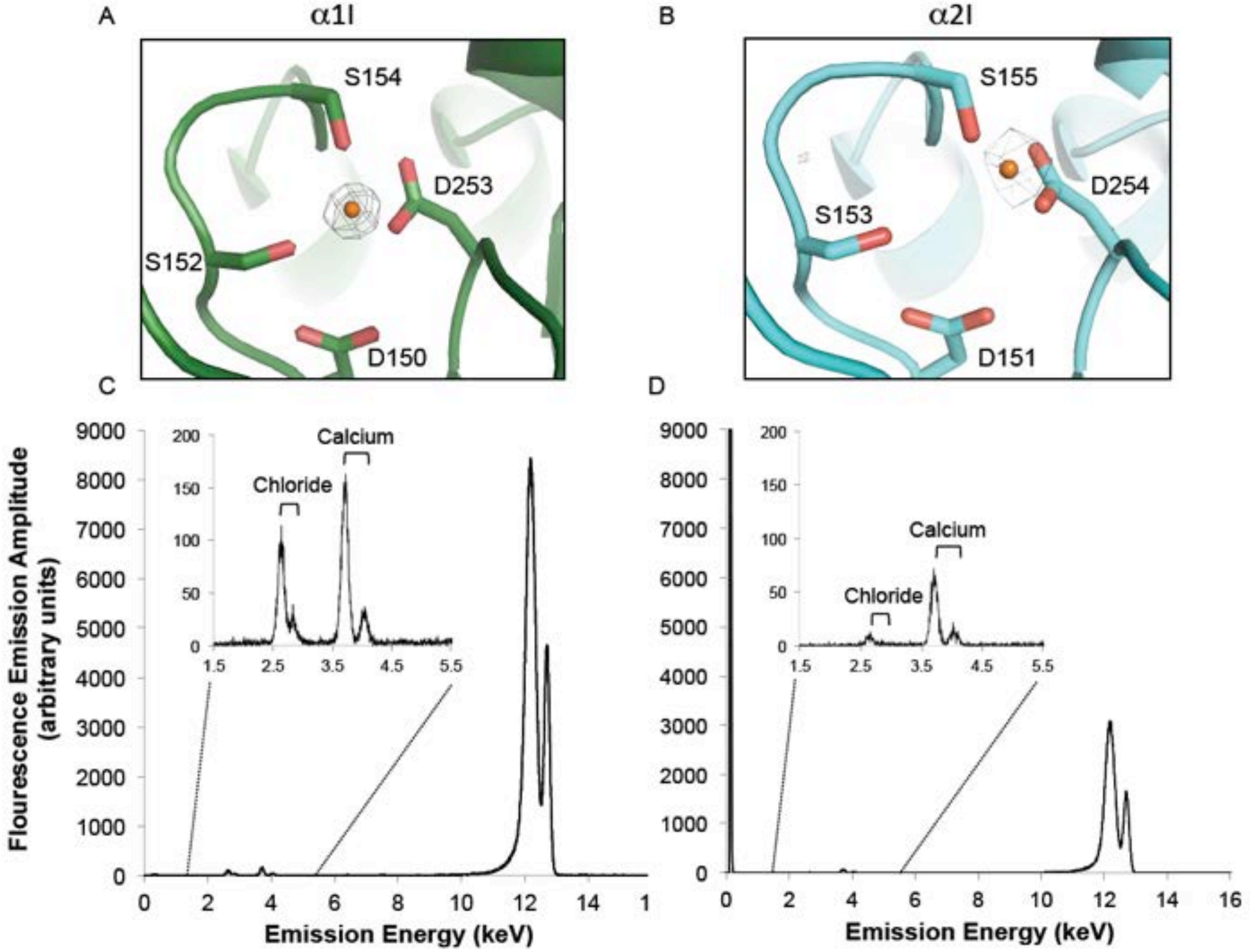

Fig. S4: Ca^{2+} is identified as the metal in the MIDAS of α1I and α2I. Anomalous difference maps from data collected above the Ca^{2+} absorption edge, contoured at 5 sigma level for α1I (A) and 3 sigma level for α2I (B). Energy dispersion X-ray spectroscopy (EDS) data was collected for α1I (C) and α2I (D) crystals. The identities of significant fluorescent emission peaks are as follows: 2.61 keV, Cl Kα1; 2.82 keV, Cl Kβ1; 3.70 keV, Ca Kα1; 4.01 keV, Ca Kβ1; 12.15 keV, Compton scattered X-rays; 12.67 keV, incident X-rays. The insets are magnifications of Ca and Cl fluorescence regions.

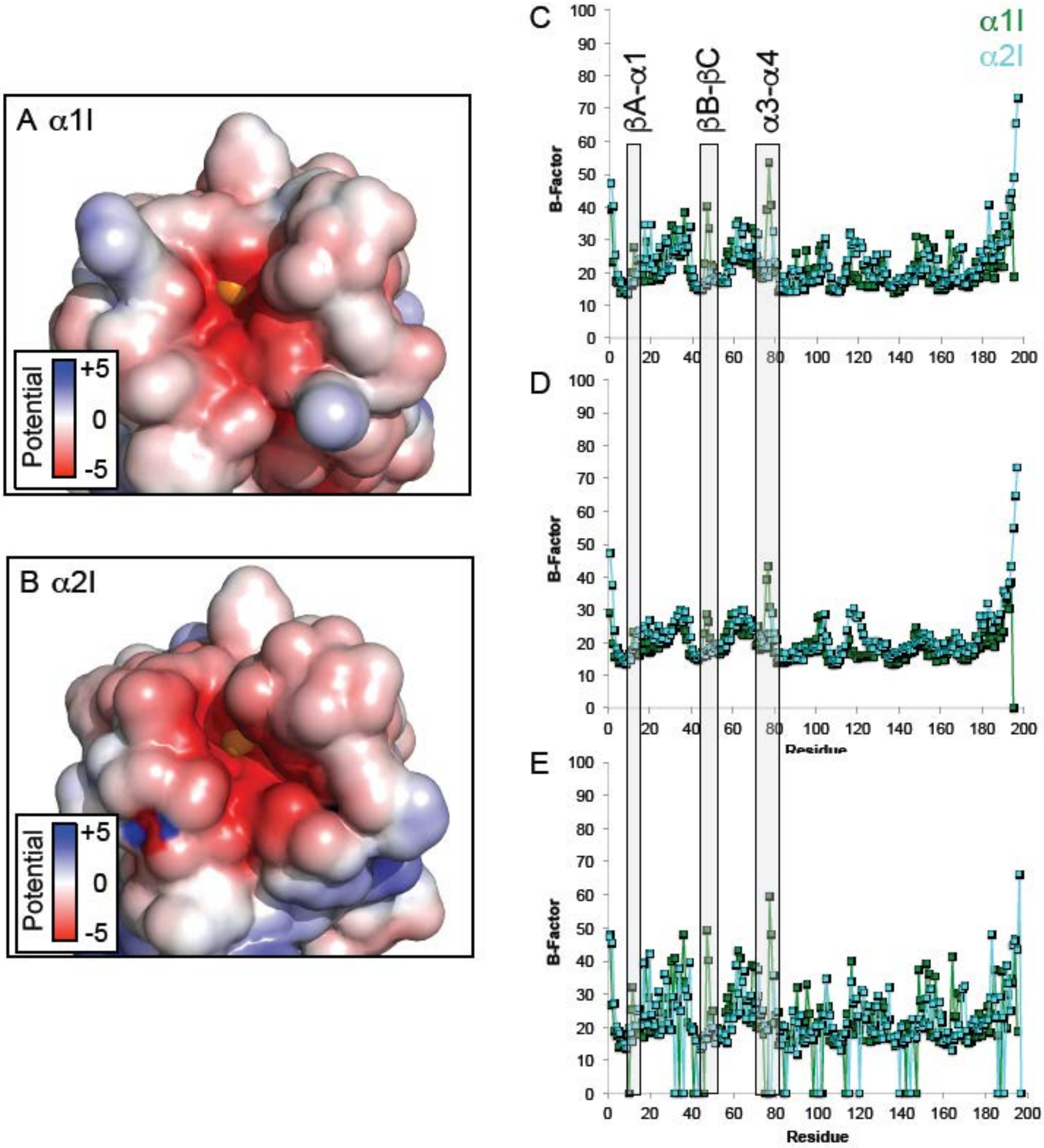

Fig. S5: Supplemental I-domain structural comparisons. The Ca^{2+}-bound α1I (A) and α2I domain (B) electrostatic surface potentials are colorimetrically depicted as a solvent accessible surface (units = Boltzman's constant (k) x temperature (298 K) / electron charge (q)). The Ca^{2+} ion appears as an orange sphere. B-factors for the Ca^{2+}-bound α1I (green) and α2I (cyan) are graphed as the average of main and side chain together (C), main chain only (D) and side chain only (E).

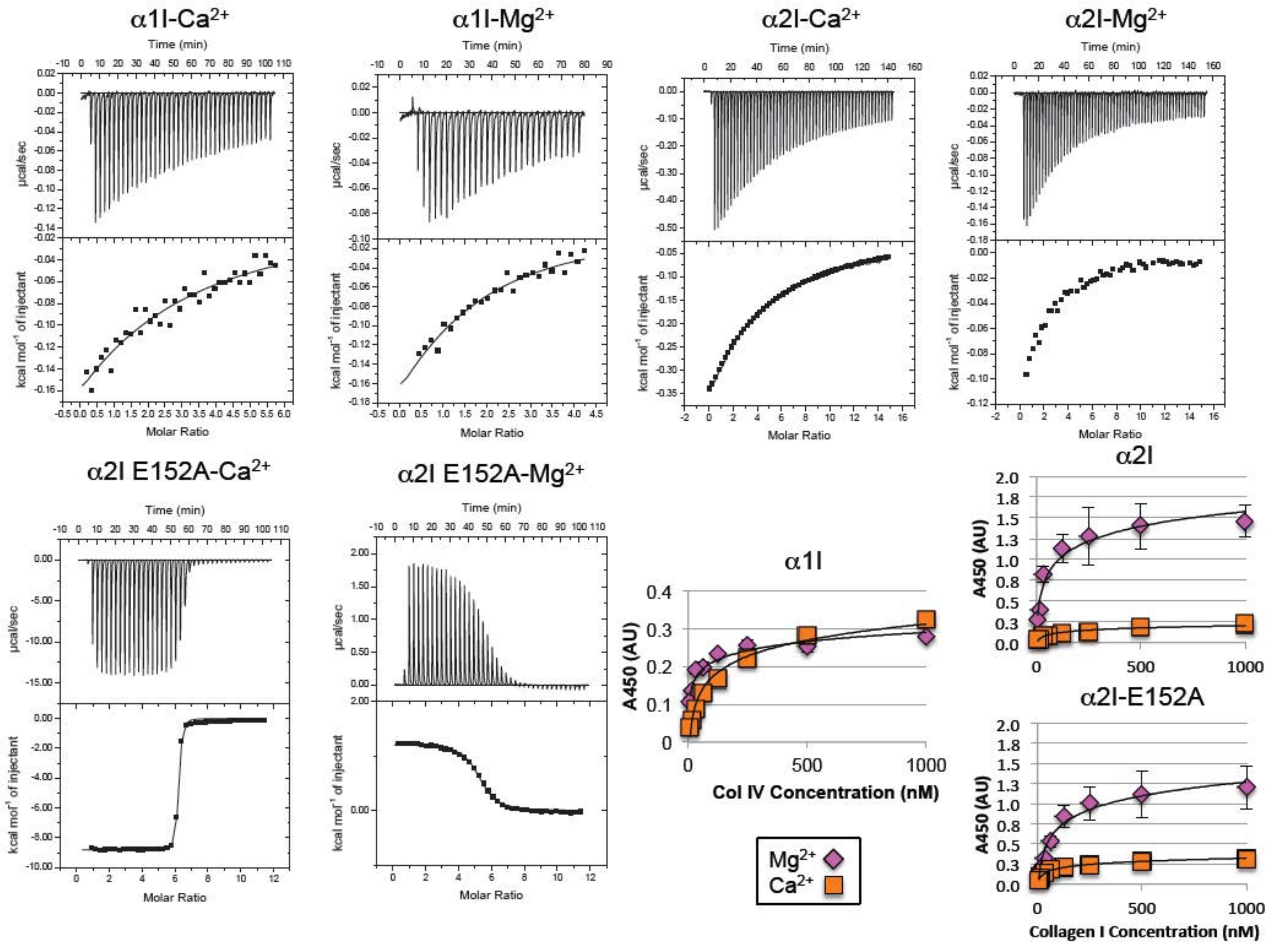

Fig. S6: Representative ITC thermograms and solid-phase binding plots. The thermodynamics of Ca^{2+} and Mg^{2+} binding to recombinant I-domains were measured by ITC. The affect of Ca^{2+} and Mg^{2+} on recombinant I-domain binding to immobilized collagen was measured with solid phase binding assays. Data are mean ± S.E (n ≥ 3).
